# Capturing Emerging Experiential Knowledge for Vaccination Guidelines Through Natural Language Processing: Proof-of-Concept Study

**DOI:** 10.2196/44461

**Published:** 2023-09-14

**Authors:** Lea Lösch, Teun Zuiderent-Jerak, Florian Kunneman, Elena Syurina, Marloes Bongers, Mart L Stein, Michelle Chan, Willemine Willems, Aura Timen

**Affiliations:** 1 Athena Institute, Faculty of Science Vrije Universiteit Amsterdam Amsterdam Netherlands; 2 Department of Computer Science, Faculty of Science Vrije Universiteit Amsterdam Amsterdam Netherlands; 3 Centre for Infectious Disease Control (CIb) National Institute for Public Health and the Environment (RIVM) Bilthoven Netherlands; 4 Department of Primary and Community Care Radboud University Medical Centre Nijmegen Netherlands

**Keywords:** guidelines as topic, COVID-19, public health, natural language processing, NLP, social media, stakeholder engagement, vaccine, vaccination, health policy, coronavirus, SARS-CoV-2

## Abstract

**Background:**

Experience-based knowledge and value considerations of health professionals, citizens, and patients are essential to formulate public health and clinical guidelines that are relevant and applicable to medical practice. Conventional methods for incorporating such knowledge into guideline development often involve a limited number of representatives and are considered to be time-consuming. Including experiential knowledge can be crucial during rapid guidance production in response to a pandemic but it is difficult to accomplish.

**Objective:**

This proof-of-concept study explored the potential of artificial intelligence (AI)–based methods to capture experiential knowledge and value considerations from existing data channels to make these insights available for public health guideline development.

**Methods:**

We developed and examined AI-based methods in relation to the COVID-19 vaccination guideline development in the Netherlands. We analyzed Dutch messages shared between December 2020 and June 2021 on social media and on 2 databases from the Dutch National Institute for Public Health and the Environment (RIVM), where experiences and questions regarding COVID-19 vaccination are reported. First, natural language processing (NLP) filtering techniques and an initial supervised machine learning model were developed to identify this type of knowledge in a large data set. Subsequently, structural topic modeling was performed to discern thematic patterns related to experiences with COVID-19 vaccination.

**Results:**

NLP methods proved to be able to identify and analyze experience-based knowledge and value considerations in large data sets. They provide insights into a variety of experiential knowledge that is difficult to obtain otherwise for rapid guideline development. Some topics addressed by citizens, patients, and professionals can serve as direct feedback to recommendations in the guideline. For example, a topic pointed out that although *travel* was not considered as a reason warranting prioritization for vaccination in the national vaccination campaign, there was a considerable need for vaccines for indispensable travel, such as cross-border informal caregiving, work or study, or accessing specialized care abroad. Another example is the ambiguity regarding the definition of medical risk groups prioritized for vaccination, with many citizens not meeting the formal priority criteria while being equally at risk. Such experiential knowledge may help the early identification of problems with the guideline’s application and point to frequently occurring exceptions that might initiate a revision of the guideline text.

**Conclusions:**

This proof-of-concept study presents NLP methods as viable tools to access and use experience-based knowledge and value considerations, possibly contributing to robust, equitable, and applicable guidelines. They offer a way for guideline developers to gain insights into health professionals, citizens, and patients’ experience-based knowledge, especially when conventional methods are difficult to implement. AI-based methods can thus broaden the evidence and knowledge base available for rapid guideline development and may therefore be considered as an important addition to the toolbox of pandemic preparedness.

## Introduction

Expert opinions and patient experiences have been deemed an essential part of evidence-based medicine right from the outset [1], as these have proved to contribute to high-quality and more applicable public health and clinical practice guidelines [2,3]. Guidelines are developed to systematically synthesize the best available evidence on a given condition, disease, or procedure, to provide recommendations that support health professionals in (clinical) decision-making. Guideline recommendations that consider experiential knowledge and patient preferences more closely reflect the needs and experiences of patients and health care professionals and thereby improve guideline adherence and patient care [4].

However, methods to achieve this, such as surveys, focus groups, and commentary on guideline drafts, vary widely, and incorporating this type of knowledge on a regular basis is not yet common practice [5-7]. Thus, there is substantial underrepresentation of experience-based knowledge and value considerations in most guidelines for a wide range of clinical topics [8].

Integrating this type of knowledge into guideline development is even more important, yet more difficult to achieve, when developing public health guidelines to respond to an ongoing pandemic. Given the urgency and high time pressure to produce the best guidance available, most guideline developers stated that prevailing methods—including those for involving end users—were largely unsuited for developing guidance during the COVID-19 pandemic [9]. In the absence of evidence from randomized clinical trials, experiential knowledge becomes one of the few sources of rapidly evolving knowledge available in the early stages of a health crisis [10,11]. The methodological challenge of its inclusion in outbreak guidance is thus an acute problem for response strategies.

The limited ability to incorporate this type of knowledge is not owing to its absence in public debate. The COVID-19 pandemic has been characterized by the extensive exchange of concerns, experiences, and value deliberations among individuals and groups, fueled by social media and the high turnover of news reports. Experiential knowledge about the subject is thus available, albeit scattered throughout media platforms and obfuscated by echo chamber characteristics of other posts on social media [12,13]. Its sheer volume and unstructured nature make it nearly impossible for guideline developers, without specific tools and methodologies, to use the experiential knowledge and value considerations of patients and professionals: out of 188 guidelines related to COVID-19 analyzed by Stamm et al [14], only 1 had involved patient knowledge.

Computational methods may offer innovative opportunities in guidance production to analyze and use existing data sources that contain experiential knowledge and value consideration systematically and on a large scale, not only after but also alongside the process of guideline development and appraisal of new information. Over the past 2 decades, studies under the heading of infodemiology have explored the use of various artificial intelligence (AI)–based methods to gain insights into disease patterns and health dynamics from digital data to inform public health and public policy [15,16]. These methods have also been further developed and used in a variety of ways across the globe for public health responses during the COVID-19 pandemic (for comprehensive reviews, refer to the papers by Syrowatka et al [17], Tsao et al [18], Chen et al [19], and Gunasekeran et al [20]). For instance, studies have aimed to provide timely and effective support to health authorities with respect to surveillance [21,22], dissemination of health information [23,24], disease detection and prediction [25,26], and monitoring of public opinion and sentiment [27,28]. Although attitudes and opinions, especially positive and negative sentiments toward COVID-19 vaccination and policies, have been analyzed extensively, exploring experiential knowledge using computational methods has received less attention (for notable exceptions, refer to the papers by Chiavi et al [29] and Bacsu et al [30]). Despite the wealth of studies exploring the applications of AI-based methods in various public health and health care contexts, automated approaches have only been explored to a limited extent in the field of guideline development, for example, to support and accelerate literature screening [31,32] but have not yet been leveraged to harness experiences as evidence for clinical or public health guidelines.

The objective of this proof-of-concept study was to develop AI methods from the field of natural language processing (NLP), for harvesting experience-based knowledge and value considerations to make guidelines more inclusive and representative and, ultimately, improve their performance in the field. Thus, our fundamental question was the following: How can AI-based methods be used to identify and analyze the experiential knowledge of health care professionals, patients, and citizens that is being shared on the web, to contribute to the development of public health guidelines? We examined these methods in the case of the development of the COVID-19 vaccination guideline in the Netherlands, which was first published in December 2020 and has since been updated in >50 guideline development and 20 user feedback meetings (as of November 2022). The guideline supports health professionals in the implementation and administration of COVID-19 vaccination [33]. It contains, for example, information about contraindications, available vaccines, and organizational and practical aspects.

## Methods

### Data Sources

We focused on 2 types of existing textual data sources: social media platforms and internal databases from the Dutch National Institute for Public Health and the Environment (RIVM). Social media were considered to be valuable for sourcing a wide range of experiences and values, as these tend to be expressed through the comment-oriented nature of posts. Facebook comments to Dutch news articles about COVID-19 vaccination were selected as a promising open data source because comments were extensive, diverse, and accessible owing to the public status of these Facebook pages through the Facebook API. In total, we collected 230,863 Facebook comments about news articles posted by the 4 popular Dutch news outlets, *Nederlandse Omroep Stichting*, *NU.nl*, *Telegraaf,* and *Nieuwe Rotterdamsche Courant*, between December 1, 2020, and June 8, 2021.

To also enable the sourcing of more targeted questions and concerns, we used 2 databases of RIVM: *InfoPunt*, the telephone and email support service for COVID-19 vaccination, and Casuïstiek Registratie Infectiezieken–operating system (*CRIos*), a case registry related to infectious diseases where professionals can report any challenges, questions, medical complications, and so on. From both databases, reports were extracted from January 2021 to June 2021 on the topic of “COVID-19 vaccination”: 34,243 anonymized emails sent to InfoPunt by citizens and professionals and 1408 from CRIos. To safeguard protected health information and to guarantee the anonymity of all senders, a data protection protocol and anonymization script were developed and applied.

### Analyses

#### Overview

**Figure 1:**
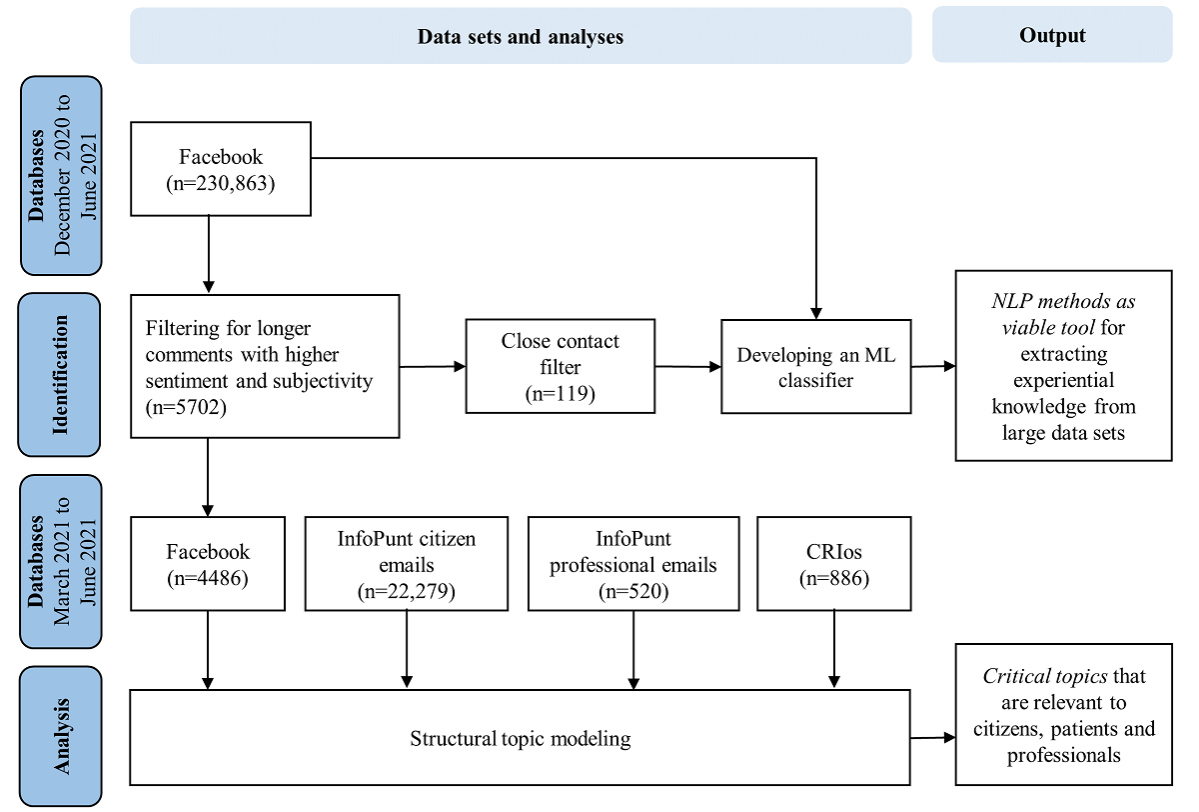
Flowchart visualizing the data analysis streams and respective outputs. CRIos: Casuïstiek Registratie Infectiezieken–operating system; ML: machine learning; NLP: natural language processing.

An impeding factor for analyzing COVID-19 vaccination experiences from social media is the predominance of expressions of attitudes and opinions in the data and that only a fraction contains descriptions of experiences. Advancing the methodology for filtering out the health information needed and making it available to responders has been identified as a major research challenge to leveraging social media for public health emergencies [34]. Therefore, to identify experiential knowledge amidst the huge volume of Facebook data, we first developed a rudimentary filter and trained a machine learning (ML) classifier. Subsequently, the selected texts and the RIVM data sets were analyzed using structural topic modeling (STM) to discover the content of people’s experiences and values related to COVID-19 vaccination. Figure 1 illustrates these data analysis streams.

#### Identifying Experience-Based Knowledge and Value Considerations

A rudimental filter was developed for the Facebook data set to narrow down the parts of the data that were likely to include experiences. First, we selected comments exceeding 250 characters, as experiences tend to be lengthy to describe. Second, posts with higher degree of subjectivity, typical for accounts of experiences and, third, posts of higher sentiment, that is, expressing stronger positive or negative emotions, were retained. To select such comments, sentiment analysis was conducted using the Python package, *Pattern* [35]. All comments were thereby assigned a value between −1 (negative) and +1 (positive) for their respective sentiment and a value between 0 (objective) and 1 (subjective) for subjectivity. Selected comments had an above-average subjectivity score (≥0.4) and a sentiment score ≤−0.25 or ≥0.25. This resulted in 5702 comments, from which a random sample of 500 (8.77%) comments was coded independently by 2 researchers for the presence of experiential knowledge (Cohen κ=0.66). The definition of experience-based knowledge related to COVID-19 vaccination in comments was kept broad, including first-hand and second-hand experiences. Our understanding is closest to the definition of *experience* from The Oxford Pocket Dictionary of Current English as “practical contact with and observation of facts or events.” We then analyzed the comments coded as experience to find further features characteristic of these comments. These descriptions often contained references to a close contact, such as a family member. This was incorporated into the filter by additionally filtering for the combination of the words “my” and a close contact, for example, “my grandma.” Whether the resulting comments exhibited experiential knowledge was assessed by another annotation round by the same 2 annotators at a moderate interannotator agreement (Cohen κ=0.60).

To detect a more diverse set of experiences beyond the rule-based filters, we set out to train a ML classifier on part of the initial data set. The “close contact” filter described previously supplied examples labeled as experiences for training the classifier. To obtain more experience posts to train on, this filter was expanded with 2 additional filter rules: the pattern, “have...had,” and a first-level or second-level hypernym to the verb, “feel” (extracted from Open Dutch Wordnet [36]).

We sampled 70,830 Facebook posts as training data from the initial set, of which 1258 (1.78%) were labeled as experience based on the 3 conditions. The posts were first cleaned of URLs, emojis, punctuation, numbers, and symbols, and the remaining tokens were lowercased. The cleaned and normalized texts were tokenized and lemmatized using the Dutch Stanza pipeline (Stanford NLP Group) [37]. Comments matching any of the 3 filters formed the baseline against which we tested the performance of 2 different algorithms, logistic regression and extreme gradient boosting [38], and 2 different feature weightings, tf*idf [39] and binary.

The best-performing model was applied to identify experiences in a heldout set of 49,034 posts. We then evaluated the number of identified experiences by inspecting 2 samples of 250 posts each, 1 with high (>0.90) and 1 with low (0.50-0.90) classifier confidence.

#### Analyzing Experience-Based Knowledge and Value Considerations

We analyzed all data sets using STM to discern thematic patterns that may relate to experiential knowledge. Topic modeling algorithms are “unsupervised” ML methods for discovering manifest and latent topics in large collections of texts [40]. “Topics” are formed based on the co-occurrence of certain words. The underlying linguistic assumption is that words that systematically appear together across multiple texts are also associated thematically [41]. Topic modeling is particularly suitable for analyzing data sets after initial relevance filtering, owing to its exploratory perspective and ability to provide rich insights into the nature of a corpus [42].

Overall, 4 different topic models were estimated. To learn about health professionals’ experiences and values related to COVID-19 vaccination, a topic model was run with 520 emails from health professionals received by InfoPunt and a second model was run with the 886 requests submitted to CRIos. To explore the experiences of citizens and patients, a third model with 22,279 emails from citizens to InfoPunt and a fourth model with 4486 long (>200 characters) comments from the high sentiment and subjectivity Facebook subset were run. Although we used only Facebook comments that resulted from the rudimentary filter, the RIVM databases did not require initial filtering; approximately all entries were regarding COVID-19 vaccination.

In all models, we included data from March 2021 onward, when vaccination of the wide population got underway in the Netherlands [43]. All non-Dutch contributions were excluded, and all texts were subjected to common preprocessing steps such as conversion to lowercase and removal of duplicates, numbers, punctuation, symbols, and web links. Words were stemmed, and a list of common Dutch “stop words,” supplied by the *quanteda* R package, was removed. In the CRIos data set, staff-specific abbreviations such as “pat” for “patient” were also omitted as certain spellings distorted the topic formation process. Finally, words that appear in very few (eg, <0.3%) or almost all (eg, >95%) documents were screened out as they are unlikely to be discriminating.

Following Roberts et al [44] we have analyzed the measures of semantic coherence and exclusivity of different models to inform the selection of an adequate number of topics (*K*). Although semantic coherence is maximized when the most probable words of a given topic occur frequently together within documents, exclusivity measures the share of top topic words that are distinct to a given topic. For the Facebook data set, the highest values for both measures were achieved at *K*=13. Similarly, this was achieved at *K*=17 for InfoPunt (citizens), *K*=8 for InfoPunt (professionals), and *K*=10 for CRIos. All topic models were conducted with the *stm* package in R [45].

To understand the resulting topics and to check the model’s validity, 20 documents highest associated with each topic were analyzed. On the basis of examining the most probable and most exclusive terms in conjunction with a close reading of exemplary documents, labels were assigned to the topics. We will provide an overview of all semantically meaningful topics; topics that are not interpretable and without substantive meaning are not presented and not displayed in the tables [46].

### Ethical Considerations

Ethics approval was not required for the analysis of the Facebook data set as it comprises publicly available posts from public pages on the platform. We did not publish specific comments that could be used to identify the original user and only share comment IDs in our Facebook data set [47] to preserve users’ privacy. The analysis of the 2 RIVM internal databases, *InfoPunt* and *CRIos*, followed a strict data protection protocol, which was compliant with the General Data Protection Regulation and approved by the RIVM privacy coordinators.

## Results

### Identifying Experience-Based Knowledge and Value Considerations

To identify experiential knowledge in Facebook data, we developed a rudimentary filter that retained long comments with high levels of sentiment and subjectivity. On the basis of the annotated set that was sampled from the filtered data, where 10% (50/500) of the messages were found to be an experience, we can assume that the same percentage holds for the entire filtered set of 5702 messages. Extending the filter by searching for comments referring to a close, personal contact resulted in 2.09% (119/5702) of comments, of which 77.3% (92/119) featured experiential knowledge.

This shows that simple but carefully selected NLP filters can identify some instances of even a fairly complex concept such as “experience” and thus greatly speed up the search for such comments. The filters also supplied examples for training an ML classifier, with the aim to capture more diverse experiences than the rule-based filters would yield.

The optimal ML setup was an extreme gradient boosting classifier using a binary weighted lemma representation, yielding an *F*_1_-score of 0.47 on predicting experiences in the annotated sample of 500 comments, considerably outperforming a rule-based filter (*F*_1_-score of 0.20). The substantial difference in recall (0.59 vs 0.14) shows the generalizability of the ML approach. After this classifier was applied to previously unseen data, 4 coders annotated samples of 250 posts with low and high classifier confidence, to evaluate the number of comments classified as experience. The coders reached a slightly weak agreement (Cohen κ=0.59). An analysis of the disagreements revealed several factors complicating the interpretation, which included nonexperiences (“My mother did not take an inoculation”), distant experiences (“I heard about people who”), and lack of context (“I did that”).

Our analysis has been a crucial step toward capturing the diversity of manifestations of experiential knowledge relevant to guideline developers, but further refinement of the ML classifier is needed before it can be integrated into the workflow of guideline developers.

### Analyzing Experience-Based Knowledge and Value Considerations

We performed STM to gain insight into the main themes addressed in different data sources by citizens (InfoPunt and Facebook) and health professionals (InfoPunt and CRIos).

#### Health Professionals’ Experiences and Values Related to COVID-19 Vaccination

The emails that health professionals directed to the InfoPunt help service revolved largely around 2 topic clusters: practical and organizational matters and questions about the vaccination of health professionals themselves (refer to Table 1 for an overview of all topics).

**Table 1:** Topics identified in health professionals’ inquiries at InfoPunt, their assigned labels, proportions, and the most frequent and simultaneously most exclusive (FREX) words (n=520).

Topic and label	Corpus, n (%)	FREX terms
1—Vaccinating other health care providers	116 (22.3)	zeneca, astra, patient, nurse, birthday, caregiver, employer, elderly, staff, and ggz
2—Business requests	100 (19.2)	registrati, zeeland, perform, ms, registered, brba, already, register, quick test, and europe
3—Administrative problems	97 (18.6)	letter, appointment, mr, client, invite, jab, mail, call, adr, and called
4—GPs^a^ and vaccination	60 (11.5)	bmi, envelopes, forwarding, forward, GPs, sir, transport, meet, online, and in advance
5—Tests	49 (9.4)	buildings, positive, tests, tested, test, symptoms, days, quarantine, scientolog, and self-test
6—Organization of vaccinations for care workers	41 (7.8)	laboratory technicians, radiological, care worker, acute, occupational group, care, contact person, infections, for example, and receives

^a^GP: general practitioner.

A topic cluster addresses administrative difficulties such as incorrect registrations of administered vaccines (topic 3) and challenges around vaccination in general practitioner (GP) practices (topic 4). Some GPs experience great strain having to organize COVID-19 vaccination in their practices in addition to their regular workload, including, for example, ordering vaccinations and prioritizing, inviting, and vaccinating patients. A second topic cluster concerns coordinating vaccination of nurses and GP practice staff who are prioritized for vaccination (topic 6). Besides, other health care professionals and employees who could not keep the recommended safe distance (1.5 m) enquire when they will get their vaccination (topic 1). This second topic cluster demonstrates how the boundary between the roles of “health professional” and “citizen” or even “patient” as adopted in our study design and in the guideline becomes blurred, as a person can fall into all 3 categories.

The questions of health professionals logged in CRIos are more technical ones (Table 2). For instance, topic 1, with the highest prevalence (150/886, 17%) in this data set, comprises reports of symptoms occurring shortly after vaccination or lasting only briefly, for example, itching skin. Another prominent subject is vaccinating patients with serious health conditions (topics 2 and 4). The guideline specifies which groups of people are prioritized for vaccination because of their health condition. However, health professionals’ questions indicate that there remains some uncertainty regarding the implementation of how (eg, with which vaccine) and by whom (eg, specialist, GP, or public health service) patients with certain conditions should be vaccinated. Moreover, professionals submit requests because some of their patients are not yet prioritized but are nevertheless deemed considerably more susceptible. This illustrates the tensions that professionals experience when they have to translate a policy that sharply defines priority groups for clear selection of patients to be prioritized for vaccination during a nationwide campaign to individualize patient care in medical practice. Another topic concerns mishaps at the vaccination location such as interrupted cold chains and vaccination with wrong needles or expired vaccines (topic 7). The analysis of topics from the case registry, owing to its focus on challenges in clinical practice, directly points out issues that arise in the application of the guideline that need to be covered and clearly explained in the guideline.

**Table 2:** Topics identified in health professionals’ inquiries at Casuïstiek Registratie Infectiezieken–operating system, their assigned labels, proportions, and the most frequent and simultaneously most exclusive (FREX) words (n=886).

Topic and label	Corpus, n (%)	FREX terms
1—Immediate vaccination reactions	150 (17)	swollen, spots, lips, feeling, itching, oedema, body, red, minutes, and itching
2—Vaccination for medical risk groups	113 (12.8)	note, friendly, hear, deemed, unfortunately, GPs^a^, want, watchman, employee, and medical
3—Pfizer vaccination	92 (10.4)	sum, other, summarize, dose, assist, birthday, madam, series, contraindication, and allergies
4—Medical conditions risk group	90 (10.2)	priority, parent, patients, quarantine, group, obesity, morbid, bmi, turn, and ml
5—Thrombosis	85 (9.6)	zeneca, 3e, thrombosis service, inr, called, agreement, astra, factor, children, and contra
6—Known allergies and other health conditions	75 (8.5)	person, jabbed, covid19, flu, reason, component, swallows, observation period, asap, and develops
7—Errors at vaccination site	74 (8.35)	resident, answer, know, hour, sure, work, guideline, logistics, two, and of course
8—Vaccination and previous COVID-19 infection	73 (8.2)	test, patient, positive, infection, scheduled, negative, antibodies, tested, past, and shorter
9—Allergic reactions	70 (7.9)	mobil, man, mg, tavegil, vaccination doctor, dd, old, diverse, day shift, and got
10—Questions and answers regarding diverse cases	64 (7.2)	account, holiday, use, website, message, url, in consultation, absolute, contraindication, and side effects

^a^GP: general practitioner.

#### Citizens’ Experiences and Values Related to COVID-19 Vaccination

Similar to professionals’ inquiries, citizens’ emails to the InfoPunt support service mainly revolved around practical questions about their COVID-19 vaccination. In total, we identified 13 technically meaningful, large topics (Table 3). A few topics were about the pandemic more broadly, for example, questions about COVID-19 preventive measures (topic 3) and criticism of policies (topic 4). However, most questions were related to practical matters. This comprises organizational, administrative questions about incorrectly addressed vaccination invitations (topic 2); vaccination certificates (topic 8); and receiving vaccination when living abroad and traveling (topic 10). Travel was not considered as a relevant reason for vaccination according to the stated national objectives of the COVID-19 vaccination campaign (prevention of disease, hospitalization, and death) and thus not addressed initially in the guideline. However, our analysis revealed that the reasons for travel were more nuanced than just for recreational purposes, resulting in a considerable need for vaccines for indispensable travel for cross-border informal caregiving, accessing specialized care in a neighboring country, or cross-border work or study.

**Table 3:** Topics identified in citizens’ inquiries at InfoPunt, their assigned labels, proportions, and the most frequent and simultaneously most exclusive (FREX) words (n=22,279).

Topic and label	Corpus, n (%)	FREX terms
1—Heterologous vaccination (AstraZeneca)	1849 (8.3)	astra, zeneca, zenica, 2nd, shot, pfizer, 1st, get, and moderna
2—Vaccination invitations	1715 (7.7)	invite, address, letter, receive, call, mail, send, present, receive, and born
3—Implementation of COVID-19 rules	1626 (7.3)	open, distance, infections, meter, measures, mask, keep, children, shop, and rule
4—Criticism of and recommendations for corona polices	1604 (7.2)	citizen, real, everyone, government, let, choice, ministry, policy, trust, and life
5—Scheduling a vaccination appointment	1603 (7.2)	appointment, call, online, download, called, make, location, succeed, and telephone
6—Thrombosis risk with AstraZeneca	1514 (6.8)	thrombosis, mother, women, birthday, afraid, father, birthday, 60-64, group, and can
7—Complaints and attachments	1470 (6.6)	sir, madam, esteemed, hereby, sir, request, awaits, organization, and dr
8—Request for vaccination certificate	1403 (6.3)	booklet, yellow, vaccination booklet, certificate, registration card, proof, registration, application, and registered
9—Definition of risk groups	1314 (5.9)	hospital, medical, patients, fall, risk group, indication, operation, asthma, priority, and care
10—Abroad	1292 (5.8)	germany, netherlands, belgium, dutch, abroad, travel, spanish, holiday, travel, and italy
11—Tests and results	1225 (5.5)	test, tested, positive, testing, pcr, result, negative, antibodies, and symptoms
12—Efficacy of different vaccines	1223 (5.5)	janssen, research, protect, variant, mrna, cases, less, effectiveness, studies, and offers
13—Vaccination side effects and second shot	1158 (5.2)	weeks, injection, two, burden, week, three, first, sensible, pain, and couple

Furthermore, questions were asked with respect to individual situations, for example, about heterologous vaccination, especially a messenger RNA vaccine following an initial shot of AstraZeneca (topic 1), and about how to proceed with the second vaccination after COVID-19 infection or adverse reactions to the first vaccination (topic 13). People are also uncertain whether they belong to the prioritized groups defined in the guideline, for example, owing to an accumulation of various mild risk factors in them (topic 9). Other people included in this prioritized group experience difficulties in receiving their vaccination, for example, because they no longer undergo active treatment but are nonetheless at risk or because their condition is not known to health professionals or authorities at all (eg, very high BMI).

What stands out in this analysis is that people who contact InfoPunt form a selection of citizens who do not seem to doubt the purpose of vaccination but already have practical questions about its implementation. As with professionals, these questions reflect the tension that might occur when applying a population-wide guideline to individual care. The RIVM InfoPunt contact center could be seen as an effort to assist in bridging this tension by providing answers to professionals’ questions when providing care for individuals. It could, with the methods presented in this paper, additionally be used for analyzing which questions arise so often that an adjusted formulation of the guideline text may be required, with more focus on such frequently occurring exceptions. The topics resulting from this analysis can thus provide insights into the issues related to individualizing guidance [3] and can indicate the possibly required guideline changes.

Citizens’ questions to InfoPunt mostly focused on the practical implementation of vaccination, whereas the Facebook comments to news articles on COVID-19 vaccination come from a more diverse group of citizens and span a wide range of values and experiences. We identified 12 technically meaningful topics (Table 4). As with InfoPunt, some topics relate to the pandemic more generally (topics 1 and 6). Among the more vaccine-specific topics, only the topic on the risk of thrombosis from vector vaccines, particularly from AstraZeneca, overlaps with those found in InfoPunt. Other topics identified in Facebook discussions revolve around uncertainties about processes and techniques that people first learned about in the wake of COVID-19 vaccination. Topic 5, for instance, assembles comments discussing the messenger RNA technique and if and how it affects the body’s DNA. Discussions also focus on how reliably vaccinations have been tested and what a vaccine’s provisional approval means (topic 11), which is often a reason for citizens to be skeptical about vaccines and consider them as actually not approved. Comments associated with topic 2 are about the purpose of vaccination, its effect on contagiousness, and the chance and severity of the COVID-19 disease.

**Table 4:** Topics identified in Facebook comments, their assigned labels, proportions, and the most frequent and simultaneously most exclusive (FREX) words (n=4486).

Topic and label	Corpus, n (%)	FREX terms
1—COVID-19 measures	489 (10.9)	freedom, mask, hear, rule, distance, holiday, oh, back, home, and shop
2—Effect of vaccination on contagiousness and disease	453 (10.1)	vaccinated, sick, infected, elderly, infecting, less, contagious, child, and serious
3—Own free choice	444 (9.9)	choice, sir, everyone, respect, own, free, fine, pressure, and feels
4—Meta-discussions	435 (9.7)	best, fine, reaction, via, message, government, latest, things, fun, and do
5—mRNA^a^ technique and consequences	354 (7.9)	term, mrna, gene, long, dna, plan, gate, compare, and effects
6—Criticism of the government	345 (7.7)	rutte, hugo, jonge, measures, cabinet, numbers, deaths, minister, bring, and ic
7—Healthy body	336 (7.5)	healthy, syringe, dead, put, inject, couple, stay, quite, junk, and trust
8—Risk of thrombosis	319 (7.1)	thrombosis, astra, pfizer, group, astrazeneca, so many, zeneca, two, pill, and janssen
9—Research	301 (6.7)	research, article, namely, israel, indeed, strong, seems, some, information, and know
10—Dangers and risks	287 (6.4)	flu, covid, dangerous, flu shot, dying, fever, scared, deadly, exists, and side effects
11—Approval and testing of the vaccination	274 (6.1)	test phase, ema, medicine, tested, 2023, package leaflet, medication, approved, error, and safe
12—Jurisdictions and costs for tests	183 (4.1)	jab, GP^b^, test, free, ggd, pcr, testing, pay, advice, and info

^a^mRNA: messenger RNA.

^b^GP: general practitioner.

This analysis of Facebook comments provides a sense of some key uncertainties that people—possibly also health professionals—express on social media about COVID-19 vaccination. Guideline users at the vaccination front line likely get confronted with these questions, to which the guideline currently does not provide answers.

The guideline provides guidance about the implementation of vaccination, such as instructions for injection, information about contraindications, vaccine combinations, and intervals [33]. An extension of the guideline by considering the uncertainties around the topics described previously could provide health professionals with possible answers to some of the public’s biggest concerns. These straightforward clarifications would potentially render the guideline more relevant and applicable for guideline users and support them more comprehensively in their day-to-day practice.

## Discussion

### Principal Findings

This paper reveals the potential of AI methods to capture experience-based knowledge and value considerations of patients, professionals, and citizens, in our case, regarding COVID-19 vaccination. We first developed strategies to filter for this type of knowledge in a large Facebook data set. We subsequently applied STM to map the landscape of questions and discussion surrounding COVID-19 vaccination in this data set and in 2 RIVM databases. Our results indicate that it is indeed possible to extract experience-based knowledge and analyze value considerations, some of which have a direct relationship to the recommendations formulated in vaccination guidelines.

By using 2 different types of data sources, we were able to identify the unique focus and added value of each. We found that people contact InfoPunt, that is, the national public health institute, for advice on practical questions about organizing their COVID-19 vaccination. The user group posting on Facebook is differently composed and has different types of questions and concerns, mainly expressed in the form of comments and prompted by the topics of the news articles. Analyzing plural data channels is hence crucial for the inclusion of a wide range of citizens and professionals’ experience-based knowledge and value considerations.

The topics revealed in the internal CRIos and InfoPunt databases are more similar to those in the guideline and thereby provide a more immediate way to incorporate value considerations and experiential knowledge in guideline development. Our finding that social media discourses are very different from what guideline developers consider relevant could have more profound implications for guideline development practices. In the initial phases of guideline development, the Population of interest, Intervention, Comparison, and Outcome framework is often consulted to select clinical questions and to search for clinical evidence. However, our analysis demonstrates that if people’s experiences and value considerations are given a more central place, one also arrives at new starting points and questions for discussion that do not fit this more clinical framing [8]. This approach also departs from public and patient involvement techniques, where these stakeholders are consulted only after scientific evidence has been gathered and priorities have been established. To improve the performance of vaccination guidelines in the field, addressing topics that are central concerns to citizens and professionals may be equally important and may need to occur during all stages of guideline development.

### Limitations and Future Studies

This analysis can be seen as a proof of concept for the analysis of experiential knowledge and values using automated text analysis methods, which yield important initial results but should be developed further. The development of our ML classifier was a crucial first step in successfully uncovering experience descriptions in a vast data set. However, the diverse manifestations of experiences make it a difficult task for an ML classifier, which is why further refinement is crucial to be able to capture experiences in large data sets with more accuracy and reliability and to yield a more complete set of experiences.

Moreover, the approach should be tested with other data sets. Regarding social media platforms, we only analyzed Facebook and did not succeed in accessing, for example, WhatsApp groups, which were used intensively to rapidly exchange information. It is thus likely that we have missed discourses on other platforms. The issue of COVID-19 vaccination presented a particular case marked by a tremendous exchange of information and experience on the web. Although this amount of data from different channels has been useful for developing our methodology, a drawback of this extent of public attention is that one must expect the deliberate spread of misinformation on the web, possibly aided by bots [48]. We tried to mitigate the impact of bots in our analysis by following a human-in-the-loop (HITL) approach, where automated analyses constantly alternated with close reading and qualitative analysis of the data. Nevertheless, our approach could be further enhanced by integrating methods specifically designed to filter out misinformation and bot-written texts [49,50].

Following this proof-of-concept study, this approach should also be extended to other (vaccine) guidelines, which are developed in less dynamic contexts. Apart from other, more widely debated topics (eg, human papillomavirus vaccination in the Netherlands), diseases that have dedicated web-based forums and those where people seek the anonymity of the internet to share their experiences could be other promising opportunities for the application of our methods in guideline development. Another challenge in method advancement is to maintain the HITL aspect while standardizing the approach. For successfully developing our approach, it was vital to constantly combine automated methods with close qualitative analysis and human assessment of the data. The HITL approach has yielded the highest diagnostic performance when using AI to support clinical practice [51]. It seems to be equally important when trying to process complex concepts such as experiential knowledge and values using automated methods. Automated methods must be flexible and sensitive to formal, linguistic, or content-related peculiarities of a given data set.

### Conclusions

Our study has shown how AI-based methods can be leveraged to extract and analyze experiential knowledge and value considerations on a large scale, which might open up new opportunities for the integration of this type of knowledge into public health guideline development, potentially improving the performance of the guidance produced. Using NLP methods, our rudimentary filter and ML classifier identified experiential knowledge in a large data set and subsequently allowed for performing STM to analyze health professionals, citizens, and patients’ experiences with COVID-19 vaccination. The methods presented offer a novel approach for guideline developers to access and gain insights into experience-based knowledge from a broad range of people, especially in cases where conventional methods of incorporating such knowledge become impractical. They thereby provide a way to broaden the evidence and knowledge base available for public health guideline development, which is particularly valuable for rapid decision-making about pandemic response strategies. Despite the limitations, this proof-of-concept study has shown that AI-based methods developed in this study may need to be considered as important additions to the toolbox of guideline development and pandemic preparedness.

## Abbreviations

AI: artificial intelligence
CRIos: Casuïstiek Registratie Infectiezieken–operating system
GP: general practitioner
HITL: human-in-the-loop
ML: machine learning
NLP: natural language processing
RIVM: Dutch National Institute for Public Health and the Environment
STM: structural topic modeling

## Data Availability

Data from the internal databases (InfoPunt and Casuïstiek Registratie Infectiezieken–operating system; CRIos) from the Dutch National Institute for Public Health and the Environment (RIVM) were supplied after anonymization under strict data protection protocols agreed between the Vrije Universiteit Amsterdam and RIVM. Owing to the sensitive nature of the data, data cannot be redistributed to researchers other than those approved in the data protection protocol, which means that the data sets are not publicly available. The data collected from Facebook are publicly available in an open data repository [47].
